# Supplementary material for: Conserved interfaces mediate multiple protein–protein interactions in a prokaryotic metabolon
Source: Mol Syst Biol. 2025 Sep 3;21(11):1490–521. doi: 10.1038/s44320-025-00139-9 (PMC12583656; doi:10.1038/s44320-025-00139-9)
Supplement: Supplementary file 1 — Appendix_file [file 44320_2025_139_MOESM1_ESM.pdf]

Appendix to “Conserved interfaces mediate multiple protein-protein interactions in a prokaryotic metabolon”

Sanchari Bhattacharyya, Srivastav Ranganathan, Sourav Chowdhury, Bharat V Adkar, Mark Khrapko and Eugene I Shakhnovich

Department of Chemistry and Chemical Biology, Harvard University, 12 Oxford St, Cambridge, MA 02138

Table of contents

Appendix_FigS1 .... Page 2

Appendix_FigS2 .... Page 3

Appendix_FigS3 .... Page 4

Appendix_FigS4 .... Page 5

Appendix_FigS5 .... Page 6

Appendix_FigS6 .... Page 7

Appendix_FigS7 .... Page 8

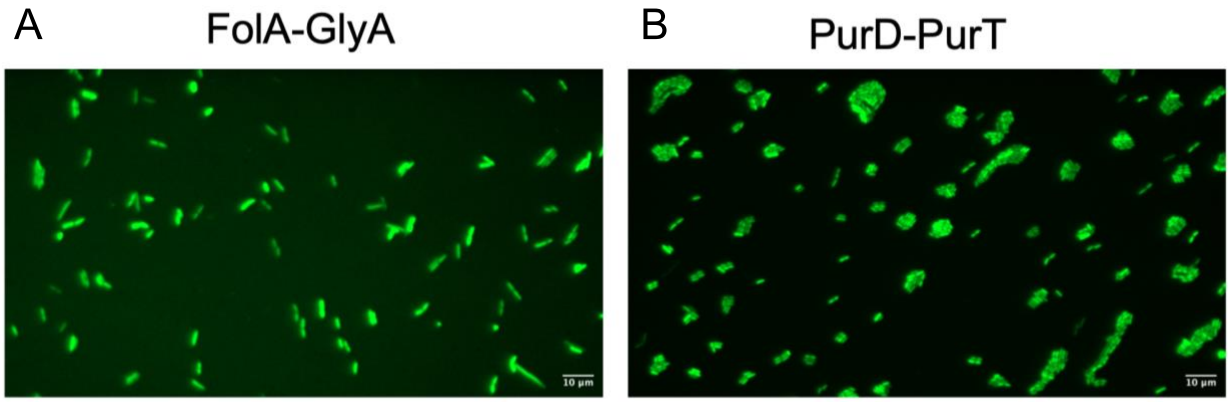

Appendix_Fig S1: Phase contrast images superimposed with YFP fluorescence of live *E. coli* cells that express (A) NYFP-FolA & CYFP-GlyA and (B) NYFP-PurD & CYFP-PurT. In both representative cases where we observe high fluorescence from FACS, the corresponding images show that YFP fluorescence is uniformly distributed inside the cells, as opposed to localization at certain areas which might signify protein aggregation.

	folE	folM	pabC	folC	pabA	nudB	folK	folA	glyA	folD	metF	purF	purD	purN	purT	purM	purK	purE	purC	purB	purH	pyrB	pyrI	pyrC	pyrD	pyrE	pyrF	pyrH	pyrG	nrdD	dcd	thyA	tmk	ndk	adk
folE	1	0.3	0.3	0.3	0.31	0.31	0.34	0.28	0.31	0.27	0.26	0.34	0.34	0.3	0.34	0.35	0.34	0.31	0.25	0.31	0.35	0.3	0.31	0.31	0.28	0.27	0.25	0.25	0.34	0.28	0.26	0.3	0.27	0.3	0.26
folM	0.3	1	0.28	0.47	0.5	0.28	0.35	0.45	0.47	0.44	0.42	0.44	0.48	0.54	0.51	0.3	0.39	0.55	0.29	0.29	0.46	0.45	0.36	0.45	0.47	0.39	0.38	0.44	0.53	0.45	0.26	0.33	0.47	0.31	0.46
pabC	0.297	0.28	1	0.37	0.3	0.27	0.35	0.27	0.33	0.28	0.25	0.35	0.3	0.33	0.33	0.31	0.33	0.29	0.31	0.23	0.33	0.28	0.43	0.3	0.29	0.31	0.28	0.3	0.33	0.33	0.27	0.29	0.29	0.32	0.26
folC	0.298	0.47	0.37	1	0.44	0.34	0.35	0.5	0.31	0.36	0.42	0.32	0.31	0.43	0.33	0.29	0.33	0.51	0.36	0.25	0.28	0.39	0.42	0.36	0.33	0.35	0.39	0.33	0.43	0.26	0.3	0.32	0.49	0.32	0.48
pabA	0.314	0.5	0.3	0.44	1	0.31	0.36	0.34	0.44	0.47	0.38	0.33	0.55	0.46	0.55	0.27	0.52	0.52	0.34	0.29	0.38	0.47	0.31	0.4	0.38	0.34	0.35	0.38	0.82	0.41	0.27	0.3	0.42	0.32	0.39
nudB	0.305	0.28	0.27	0.34	0.31	1	0.43	0.29	0.32	0.29	0.29	0.29	0.3	0.28	0.29	0.32	0.3	0.3	0.28	0.32	0.35	0.29	0.3	0.31	0.3	0.29	0.3	0.32	0.32	0.34	0.3	0.28	0.34	0.41	0.3
folK	0.344	0.35	0.35	0.35	0.36	0.43	1	0.28	0.38	0.36	0.37	0.36	0.3	0.34	0.3	0.29	0.33	0.3	0.31	0.32	0.33	0.36	0.39	0.39	0.39	0.34	0.31	0.39	0.37	0.43	0.29	0.36	0.37	0.54	0.37
folA	0.283	0.45	0.27	0.5	0.34	0.29	0.28	1	0.47	0.42	0.42	0.41	0.44	0.48	0.41	0.33	0.31	0.34	0.31	0.3	0.38	0.46	0.29	0.43	0.4	0.39	0.37	0.49	0.54	0.4	0.27	0.37	0.46	0.26	0.47
glyA	0.311	0.47	0.33	0.31	0.44	0.32	0.38	0.47	1	0.37	0.36	0.34	0.3	0.42	0.32	0.27	0.32	0.5	0.35	0.24	0.29	0.36	0.36	0.34	0.34	0.46	0.38	0.35	0.31	0.31	0.32	0.34	0.38	0.36	0.36
folD	0.266	0.44	0.28	0.36	0.47	0.29	0.36	0.42	0.37	1	0.35	0.42	0.37	0.45	0.4	0.26	0.37	0.51	0.27	0.29	0.32	0.62	0.37	0.35	0.33	0.48	0.35	0.34	0.42	0.34	0.27	0.29	0.38	0.37	0.31
metF	0.263	0.42	0.25	0.42	0.38	0.29	0.37	0.42	0.36	0.35	1	0.38	0.37	0.4	0.36	0.29	0.33	0.47	0.29	0.29	0.37	0.34	0.31	0.61	0.57	0.42	0.68	0.43	0.43	0.44	0.3	0.33	0.42	0.33	0.4
purF	0.337	0.44	0.35	0.32	0.33	0.29	0.36	0.41	0.34	0.42	0.38	1	0.35	0.38	0.34	0.29	0.34	0.37	0.31	0.24	0.26	0.38	0.4	0.36	0.33	0.57	0.38	0.35	0.32	0.28	0.29	0.33	0.39	0.36	0.33
purD	0.34	0.48	0.3	0.31	0.55	0.3	0.3	0.44	0.3	0.37	0.37	0.35	1	0.54	0.72	0.28	0.76	0.48	0.4	0.23	0.25	0.38	0.37	0.37	0.3	0.37	0.38	0.38	0.36	0.3	0.32	0.31	0.38	0.35	0.3
purN	0.299	0.54	0.33	0.43	0.46	0.28	0.34	0.48	0.42	0.45	0.4	0.38	0.54	1	0.54	0.36	0.43	0.51	0.29	0.31	0.41	0.47	0.32	0.39	0.4	0.37	0.34	0.43	0.52	0.39	0.27	0.31	0.45	0.3	0.4
purT	0.336	0.51	0.33	0.33	0.55	0.29	0.3	0.41	0.32	0.4	0.36	0.34	0.72	0.54	1	0.3	0.84	0.5	0.43	0.24	0.28	0.38	0.34	0.36	0.3	0.39	0.39	0.38	0.36	0.29	0.31	0.31	0.42	0.35	0.39
purM	0.347	0.3	0.31	0.29	0.27	0.32	0.29	0.33	0.27	0.26	0.29	0.29	0.28	0.36	0.3	1	0.28	0.28	0.29	0.28	0.28	0.28	0.36	0.27	0.25	0.32	0.25	0.32	0.3	0.27	0.29	0.26	0.28	0.44	0.26
purK	0.342	0.39	0.33	0.33	0.52	0.3	0.33	0.31	0.32	0.37	0.33	0.34	0.76	0.43	0.84	0.28	1	0.45	0.41	0.22	0.27	0.36	0.33	0.33	0.27	0.34	0.34	0.28	0.33	0.29	0.28	0.29	0.35	0.35	0.32
purE	0.308	0.55	0.29	0.51	0.52	0.3	0.3	0.34	0.5	0.51	0.47	0.37	0.48	0.51	0.5	0.28	0.45	1	0.28	0.34	0.38	0.5	0.27	0.48	0.44	0.4	0.46	0.44	0.58	0.49	0.26	0.31	0.49	0.29	0.43
purC	0.254	0.29	0.31	0.36	0.34	0.28	0.31	0.31	0.35	0.27	0.29	0.31	0.4	0.29	0.43	0.29	0.41	0.28	1	0.25	0.29	0.29	0.37	0.35	0.29	0.26	0.31	0.28	0.35	0.36	0.26	0.3	0.31	0.32	0.32
purB	0.312	0.29	0.23	0.25	0.29	0.32	0.32	0.3	0.24	0.29	0.29	0.24	0.23	0.31	0.24	0.28	0.22	0.34	0.25	1	0.26	0.26	0.33	0.22	0.25	0.27	0.32	0.3	0.25	0.26	0.29	0.26	0.32	0.33	0.29
purH	0.351	0.46	0.33	0.28	0.38	0.35	0.33	0.38	0.29	0.32	0.37	0.26	0.25	0.41	0.28	0.28	0.27	0.38	0.29	0.26	1	0.29	0.38	0.31	0.3	0.37	0.36	0.38	0.26	0.23	0.29	0.32	0.39	0.39	0.38
pyrB	0.299	0.45	0.28	0.39	0.47	0.29	0.36	0.46	0.36	0.62	0.34	0.38	0.38	0.47	0.38	0.28	0.36	0.5	0.29	0.26	0.29	1	0.4	0.31	0.31	0.43	0.38	0.35	0.41	0.35	0.29	0.29	0.37	0.3	0.37
pyrI	0.314	0.36	0.43	0.42	0.31	0.3	0.39	0.29	0.36	0.37	0.31	0.4	0.37	0.32	0.34	0.36	0.33	0.27	0.37	0.33	0.38	0.4	1	0.28	0.35	0.33	0.27	0.31	0.4	0.33	0.29	0.3	0.3	0.4	0.32
pyrC	0.306	0.45	0.3	0.36	0.4	0.31	0.39	0.43	0.34	0.35	0.61	0.36	0.37	0.39	0.36	0.27	0.33	0.48	0.35	0.22	0.31	0.31	0.28	1	0.46	0.41	0.64	0.41	0.4	0.41	0.32	0.39	0.42	0.35	0.41
pyrD	0.285	0.47	0.29	0.33	0.38	0.3	0.39	0.4	0.34	0.33	0.57	0.33	0.3	0.4	0.3	0.25	0.27	0.44	0.29	0.25	0.3	0.31	0.35	0.46	1	0.43	0.71	0.38	0.36	0.39	0.28	0.34	0.43	0.31	0.36
pyrE	0.265	0.39	0.31	0.35	0.34	0.29	0.34	0.39	0.46	0.48	0.42	0.57	0.37	0.37	0.39	0.32	0.34	0.4	0.26	0.27	0.37	0.43	0.33	0.41	0.43	1	0.37	0.43	0.4	0.36	0.28	0.29	0.31	0.31	0.3
pyrF	0.25	0.38	0.28	0.39	0.35	0.3	0.31	0.37	0.38	0.35	0.68	0.38	0.38	0.34	0.39	0.25	0.34	0.46	0.31	0.32	0.36	0.38	0.27	0.64	0.71	0.37	1	0.39	0.41	0.48	0.26	0.32	0.38	0.29	0.36
pyrH	0.25	0.44	0.3	0.33	0.38	0.32	0.39	0.49	0.35	0.34	0.43	0.35	0.38	0.43	0.38	0.32	0.28	0.44	0.28	0.3	0.38	0.35	0.31	0.41	0.38	0.43	0.39	1	0.46	0.41	0.28	0.29	0.4	0.32	0.4
pyrG	0.345	0.53	0.33	0.43	0.82	0.32	0.37	0.54	0.31	0.42	0.43	0.32	0.36	0.52	0.36	0.3	0.33	0.58	0.35	0.25	0.26	0.41	0.4	0.4	0.36	0.4	0.41	0.46	1	0.27	0.3	0.3	0.51	0.32	0.46
nrdD	0.278	0.45	0.33	0.26	0.41	0.34	0.43	0.4	0.31	0.34	0.44	0.28	0.3	0.39	0.29	0.27	0.29	0.49	0.36	0.26	0.23	0.35	0.33	0.41	0.39	0.36	0.48	0.41	0.27	1	0.34	0.32	0.45	0.42	0.41
dcd	0.256	0.26	0.27	0.3	0.27	0.3	0.29	0.27	0.32	0.27	0.3	0.29	0.32	0.27	0.31	0.29	0.28	0.26	0.26	0.29	0.29	0.29	0.29	0.32	0.28	0.28	0.26	0.28	0.3	0.34	1	0.29	0.25	0.28	0.25
thyA	0.301	0.33	0.29	0.32	0.3	0.28	0.36	0.37	0.34	0.29	0.33	0.33	0.31	0.31	0.31	0.26	0.29	0.31	0.3	0.26	0.32	0.29	0.3	0.39	0.34	0.29	0.32	0.29	0.3	0.32	0.29	1	0.41	0.29	0.36
tmk	0.272	0.47	0.29	0.49	0.42	0.34	0.37	0.46	0.38	0.38	0.42	0.39	0.38	0.45	0.42	0.28	0.35	0.49	0.31	0.32	0.39	0.37	0.3	0.42	0.43	0.31	0.38	0.4	0.51	0.45	0.25	0.41	1	0.35	0.61
ndk	0.297	0.31	0.32	0.32	0.32	0.41	0.54	0.26	0.36	0.37	0.33	0.36	0.35	0.3	0.35	0.44	0.35	0.29	0.32	0.33	0.39	0.3	0.4	0.35	0.31	0.31	0.29	0.32	0.32	0.42	0.28	0.29	0.35	1	0.37
adk	0.256	0.46	0.26	0.48	0.39	0.3	0.37	0.47	0.36	0.31	0.4	0.33	0.3	0.4	0.39	0.26	0.32	0.43	0.32	0.29	0.38	0.37	0.32	0.41	0.36	0.3	0.36	0.4	0.46	0.41	0.25	0.36	0.61	0.37	1

Appendix_Fig S2: A 35x35 matrix for TM score of each protein pair. A symmetric matrix was generated by taking the maximum value for each protein pair.

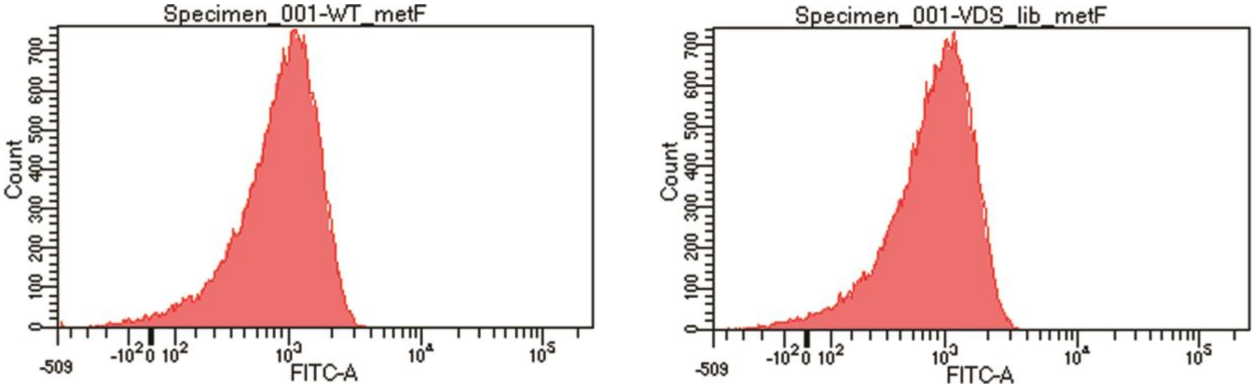

Appendix_Fig S3: Fluorescence intensity distributions of ~30,000 cells transformed with NYFP-WT DHFR/CYFP-MetF plasmids (left panel) and NYFP-VDS_lib DHFR/CYFP-MetF plasmids (right panel). The histogram obtained with VDS library does not appear to be different from the WT histogram.

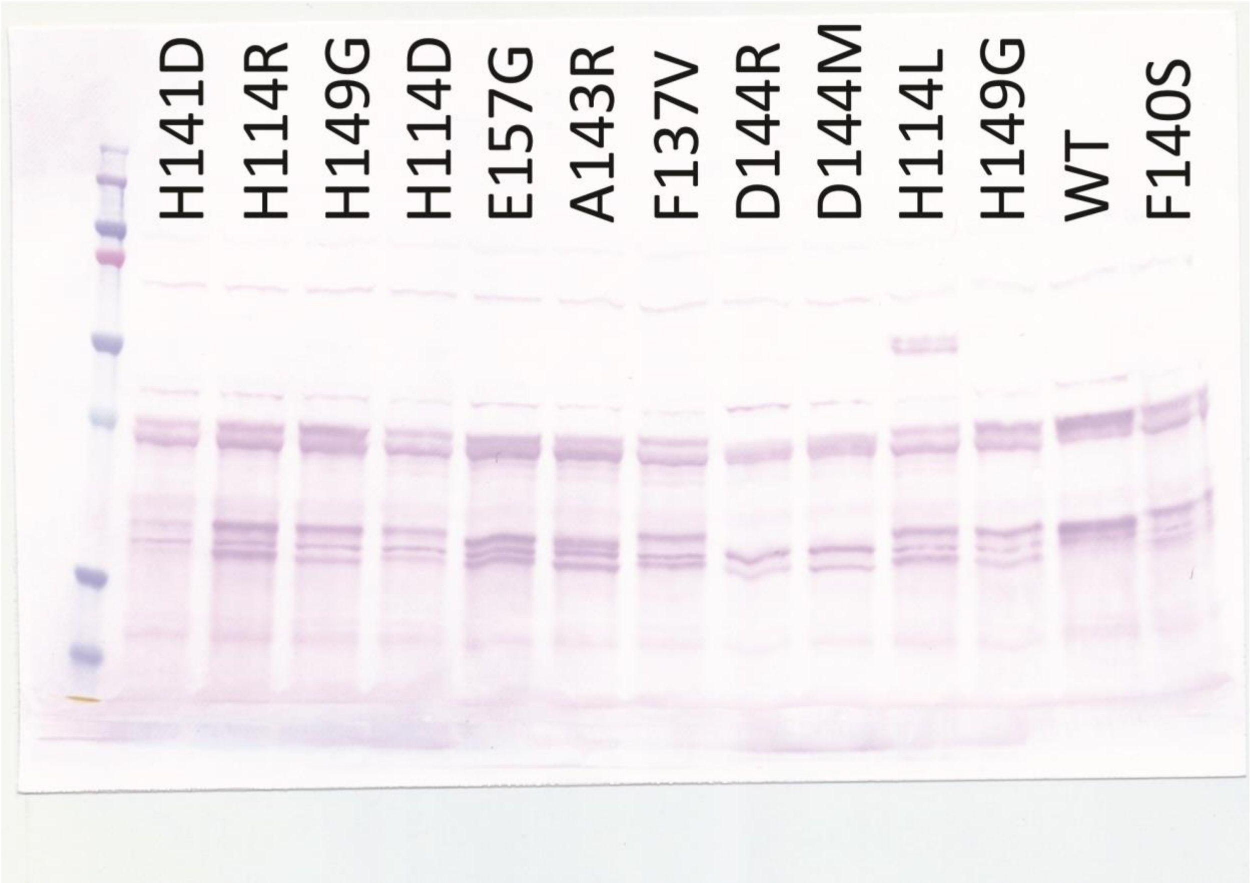

Appendix_Fig S4: Protein expression check using western blot for DHFR library variants which show substantial loss or increase in fluorescence relative to WT. No significant change in expression was observed, indicating that the change in fluorescence is not due to generic loss of protein levels.

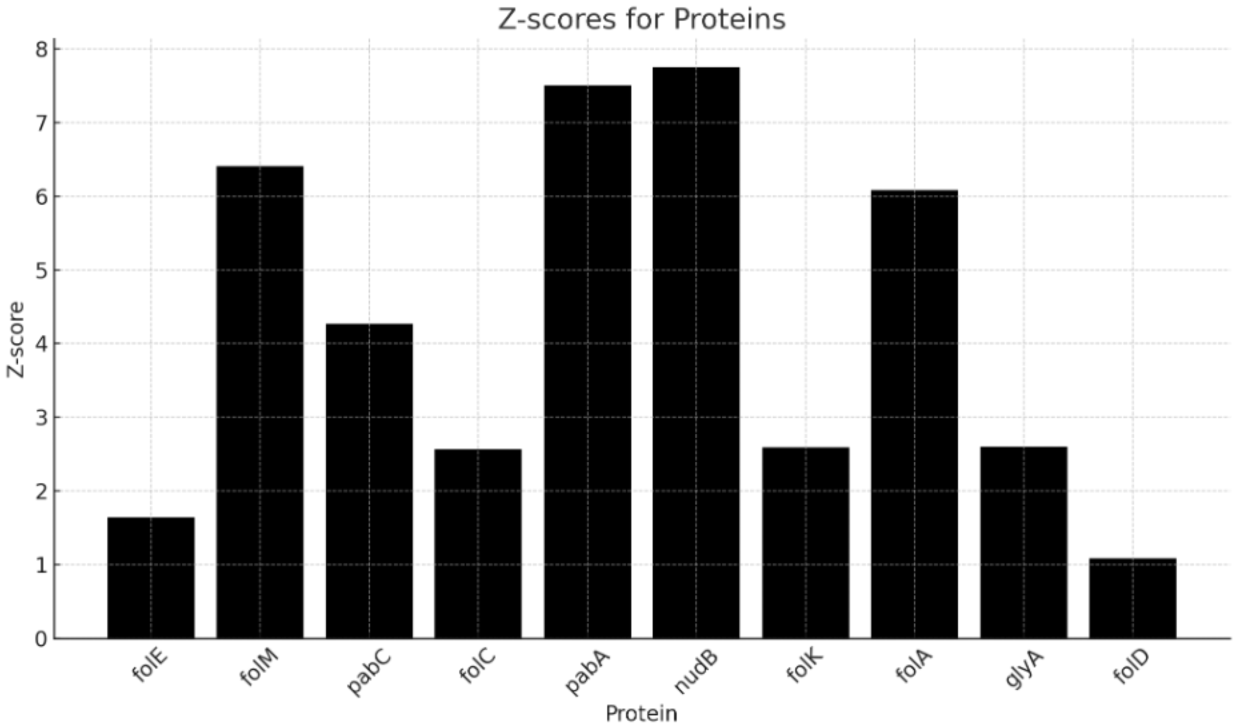

Appendix_Fig S5. Z-scores for the shift in mean inter-surface contacts (observed) compared to the randomized set. The Z score measures how many standard deviations from the mean the observed value lies and determines p-value for the underlying t-statistic for the random null model (see Methods section for details). We set a significance level (e.g., 0.05), which represents the threshold for statistical significance. If the p-value is less than this significance level, we reject the null hypothesis. Otherwise, the null hypothesis is accepted.

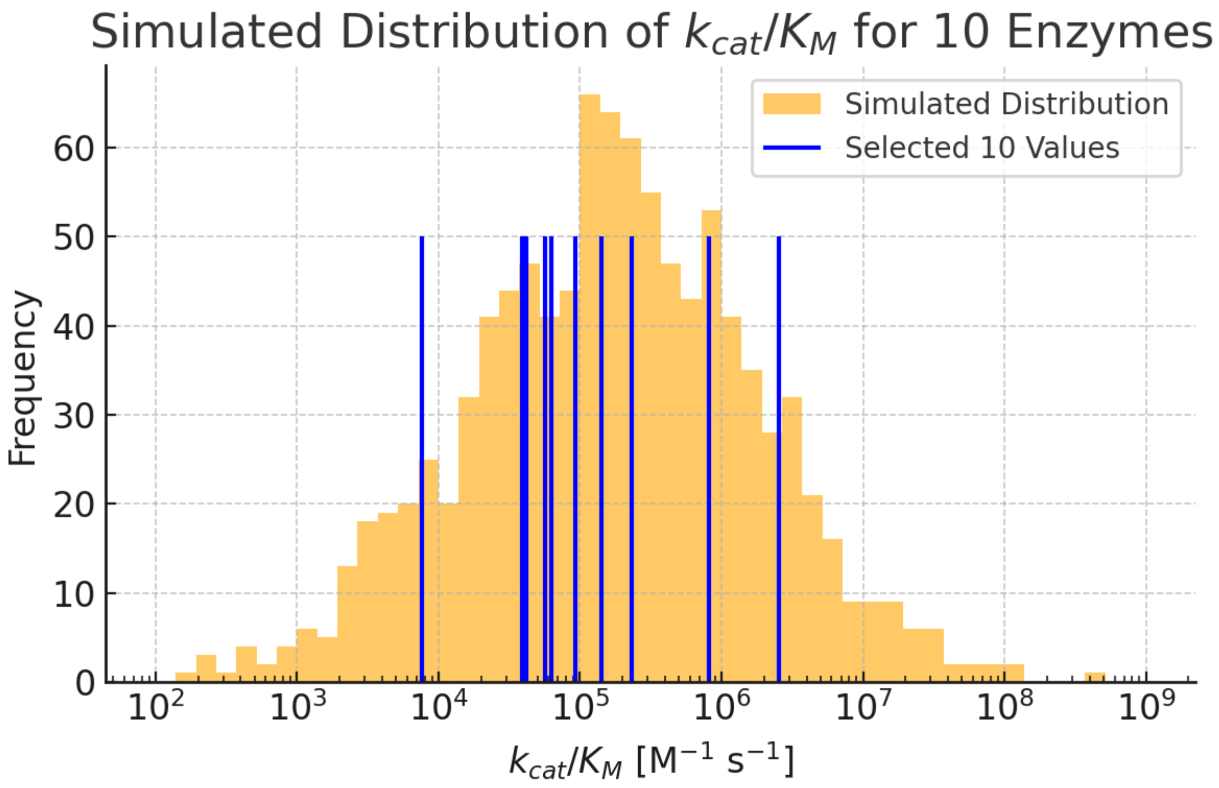

Appendix_Fig S6. The histogram (orange) represents a simulated population of natural enzyme k_{cat}/K_M values drawn from a log-normal distribution centered around 10^5 $M^{-1}s^{-1}$ with a spread of ±1 log unit, based on empirical data from biochemical databases. Blue vertical lines indicate the 10 representative enzyme values selected for the 10-step pathway modeled in the coarse-grained simulation. These values span the realistic catalytic efficiency range observed in natural enzymes, from 10^3 to 10^7 $M^{-1}s^{-1}$. The distribution of k_{cat}/K_M (orange histograms) are based on data from (Bar-Even *et al.*, 2015).

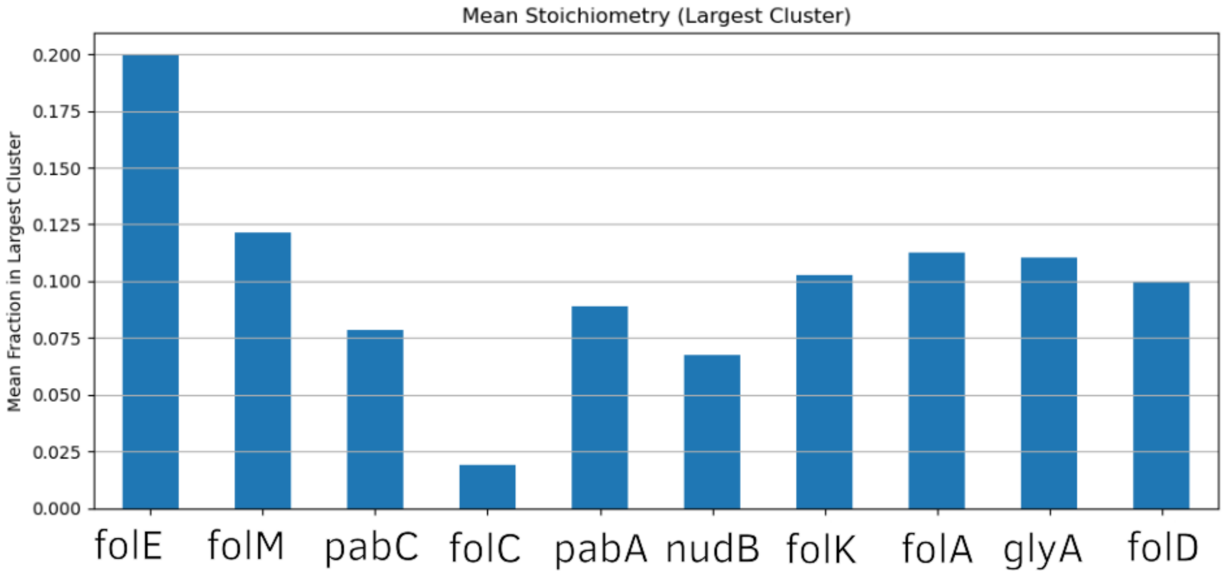

Appendix_Fig S7. Stoichiometry of the largest cluster at equilibrium. Imposing the folate pathway PPI in the Langevin dynamics simulations results in clusters that contain several copies of each enzyme.
