# Supplementary figures and images for: Conserved interfaces mediate multiple protein–protein interactions in a prokaryotic metabolon
Source: Mol Syst Biol. 2025 Sep 3;21(11):1490–521. doi: 10.1038/s44320-025-00139-9 (PMC12583656; doi:10.1038/s44320-025-00139-9)

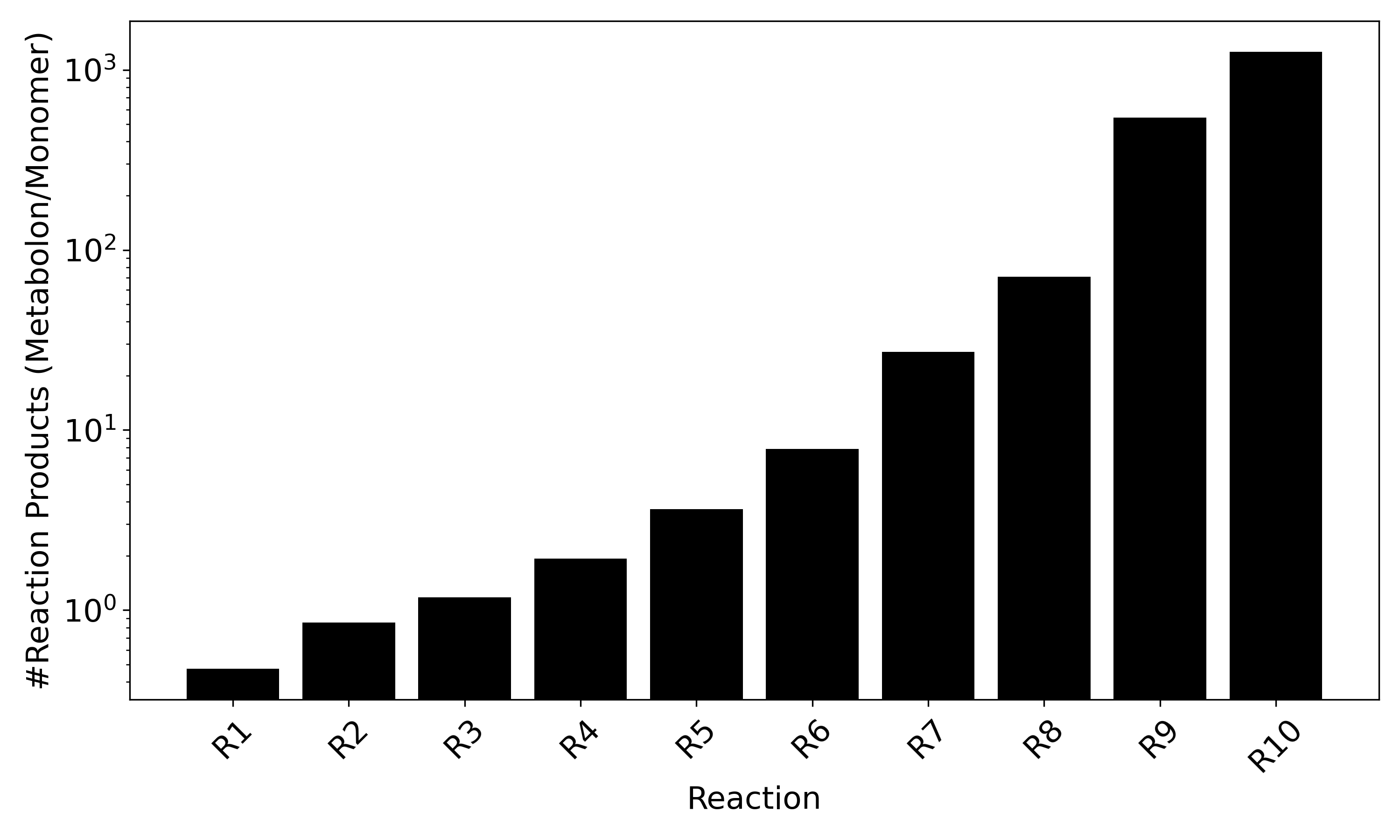

Supplement: Supplementary file 13 — Source data Fig. 8 [file 44320_2025_139_MOESM13_ESM.zip › Figure8/Simulation_software_config_to_run_sim/Disassembled/reaction_products_comparison.png]

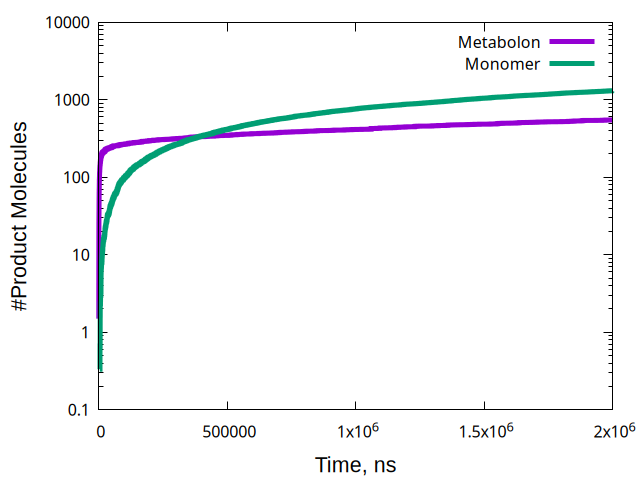

Supplement: Supplementary file 13 — Source data Fig. 8 [file 44320_2025_139_MOESM13_ESM.zip › Figure8/Simulation_software_config_to_run_sim/Disassembled/rx1_compare.png]

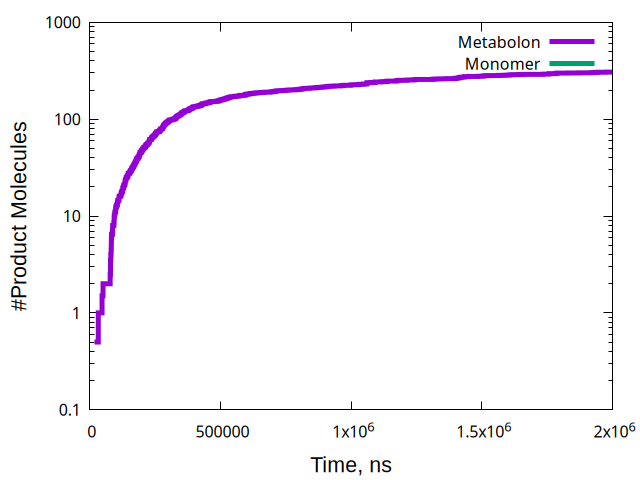

Supplement: Supplementary file 13 — Source data Fig. 8 [file 44320_2025_139_MOESM13_ESM.zip › Figure8/Simulation_software_config_to_run_sim/Disassembled/rx6_compare.png]

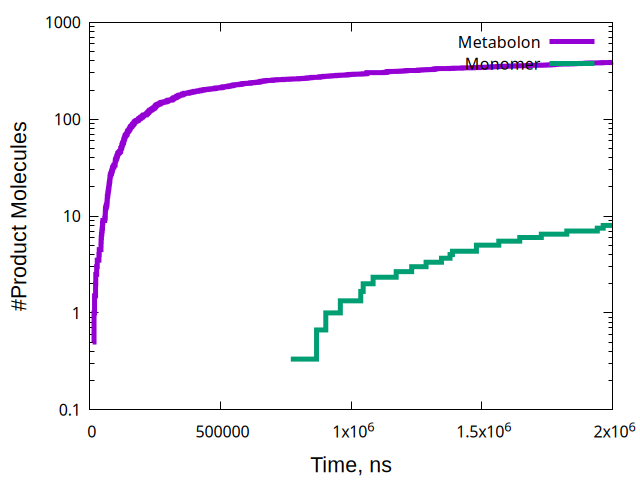

Supplement: Supplementary file 13 — Source data Fig. 8 [file 44320_2025_139_MOESM13_ESM.zip › Figure8/Simulation_software_config_to_run_sim/Disassembled/rx4_compare.png]

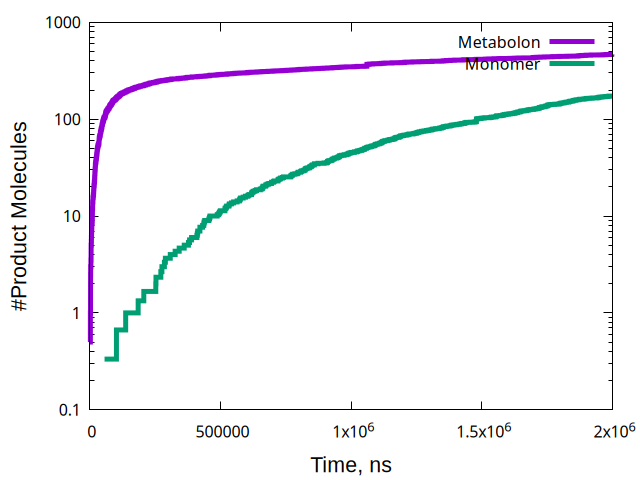

Supplement: Supplementary file 13 — Source data Fig. 8 [file 44320_2025_139_MOESM13_ESM.zip › Figure8/Simulation_software_config_to_run_sim/Disassembled/rx2_compare.png]

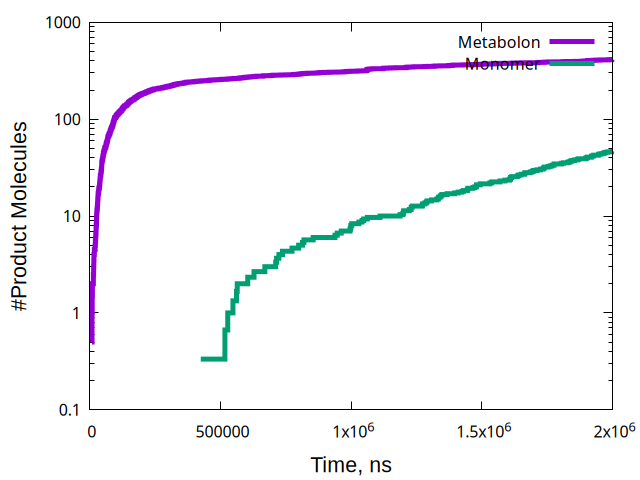

Supplement: Supplementary file 13 — Source data Fig. 8 [file 44320_2025_139_MOESM13_ESM.zip › Figure8/Simulation_software_config_to_run_sim/Disassembled/rx3_compare.png]

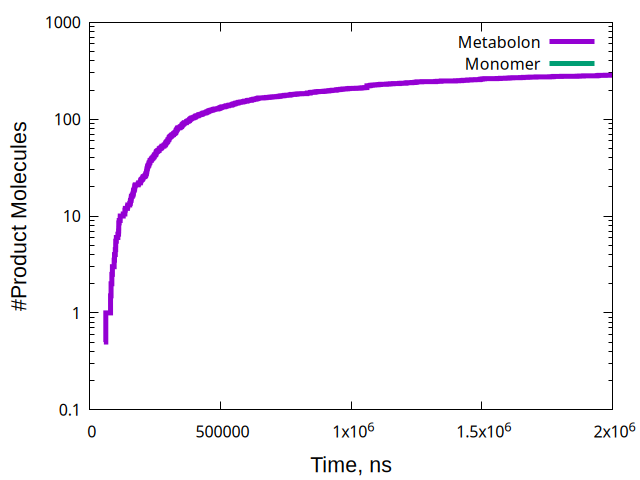

Supplement: Supplementary file 13 — Source data Fig. 8 [file 44320_2025_139_MOESM13_ESM.zip › Figure8/Simulation_software_config_to_run_sim/Disassembled/rx7_compare.png]

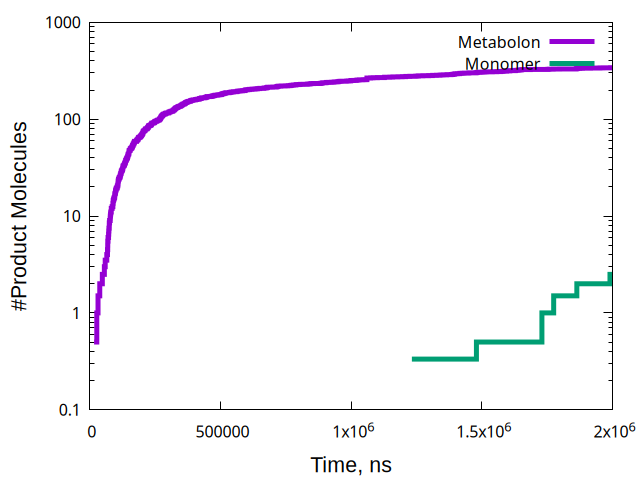

Supplement: Supplementary file 13 — Source data Fig. 8 [file 44320_2025_139_MOESM13_ESM.zip › Figure8/Simulation_software_config_to_run_sim/Disassembled/rx5_compare.png]

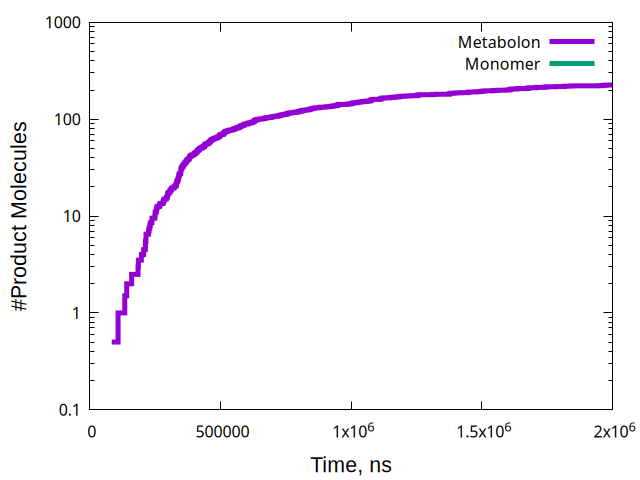

Supplement: Supplementary file 13 — Source data Fig. 8 [file 44320_2025_139_MOESM13_ESM.zip › Figure8/Simulation_software_config_to_run_sim/Disassembled/rx10_compare.png]

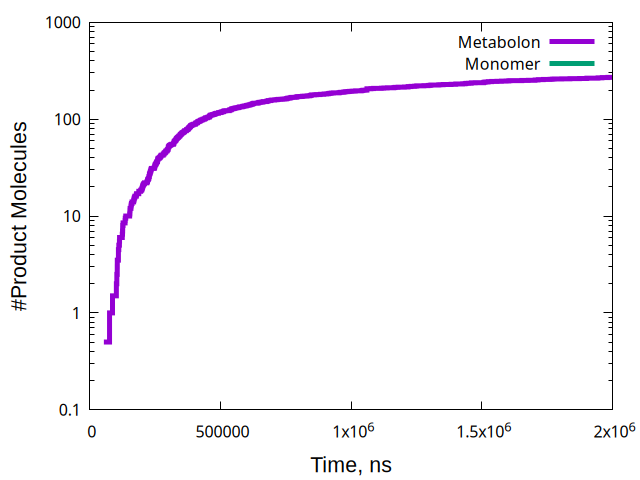

Supplement: Supplementary file 13 — Source data Fig. 8 [file 44320_2025_139_MOESM13_ESM.zip › Figure8/Simulation_software_config_to_run_sim/Disassembled/rx8_compare.png]

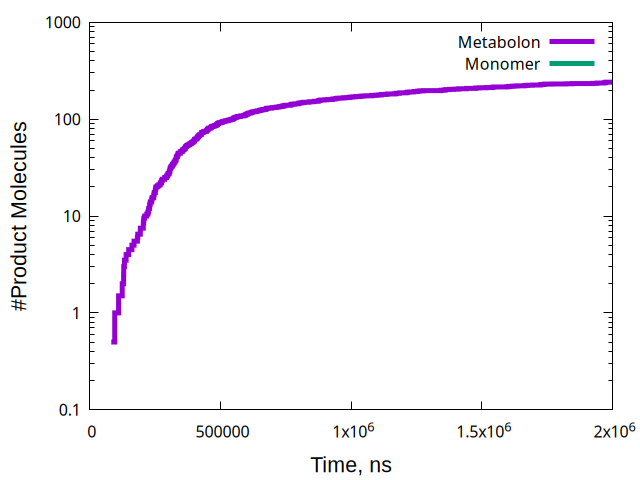

Supplement: Supplementary file 13 — Source data Fig. 8 [file 44320_2025_139_MOESM13_ESM.zip › Figure8/Simulation_software_config_to_run_sim/Disassembled/rx9_compare.png]

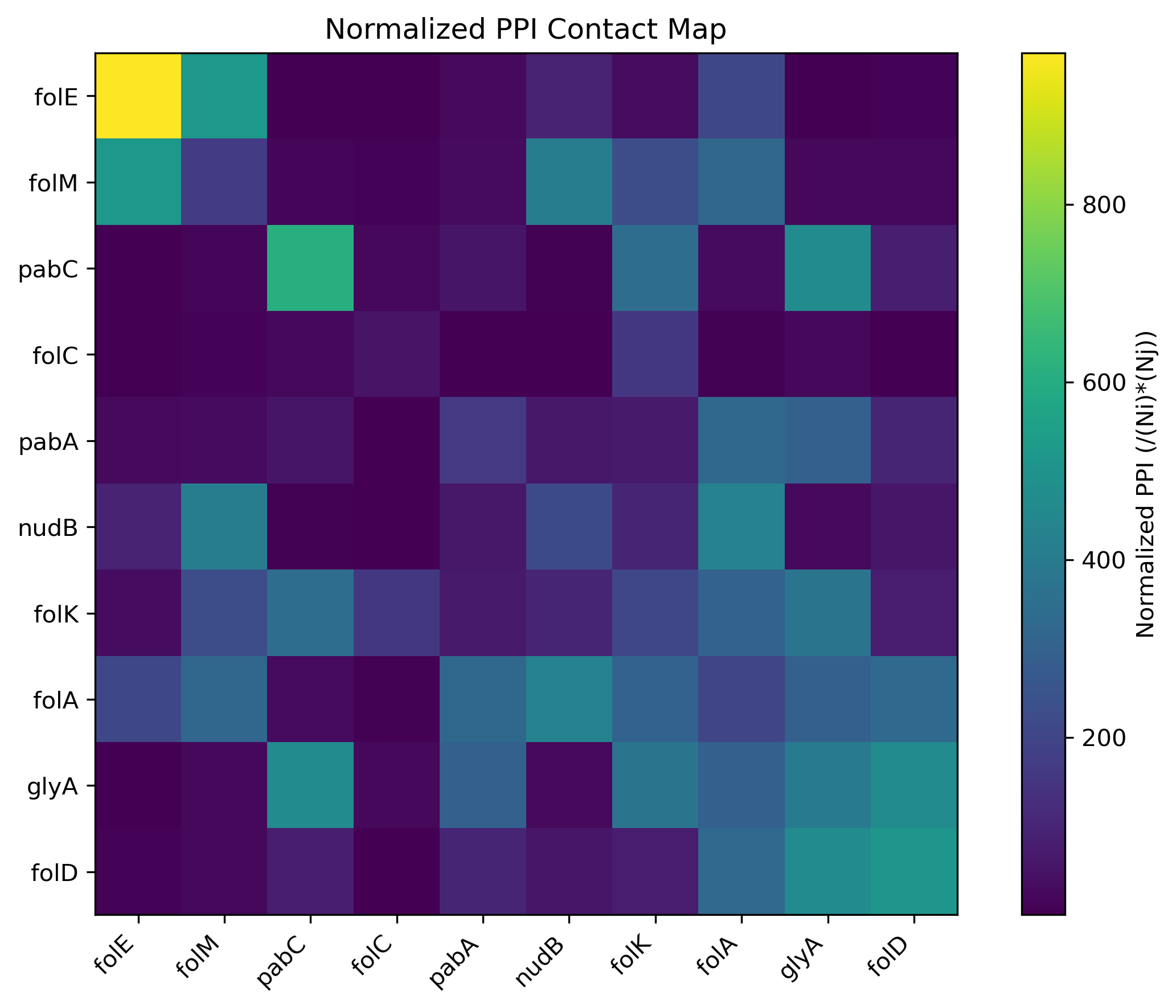

Supplement: Supplementary file 13 — Source data Fig. 8 [file 44320_2025_139_MOESM13_ESM.zip › Figure8/Simulation_software_config_to_run_sim/Metabolon/ppi_contact_map.png]

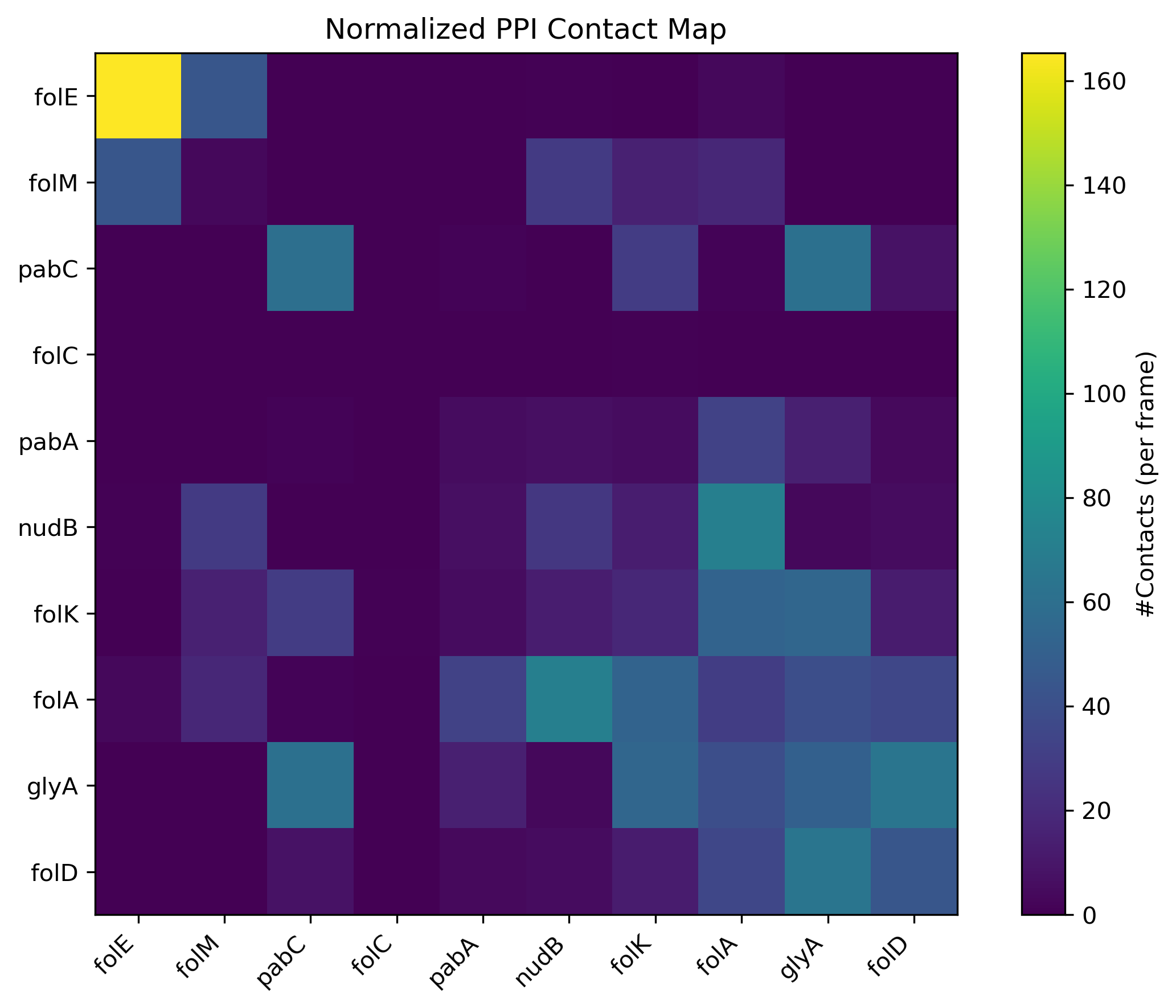

Supplement: Supplementary file 13 — Source data Fig. 8 [file 44320_2025_139_MOESM13_ESM.zip › Figure8/Simulation_software_config_to_run_sim/Metabolon/ppi_contact_map_set2.png]

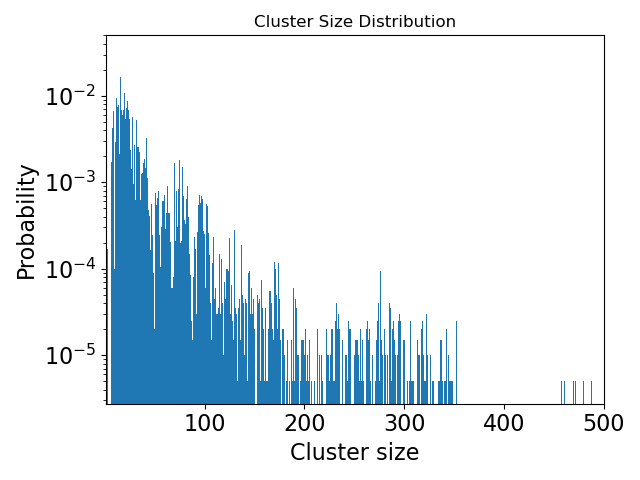

Supplement: Supplementary file 13 — Source data Fig. 8 [file 44320_2025_139_MOESM13_ESM.zip › Figure8/Simulation_software_config_to_run_sim/Metabolon/avg_cluster_distribution_second_half.png]

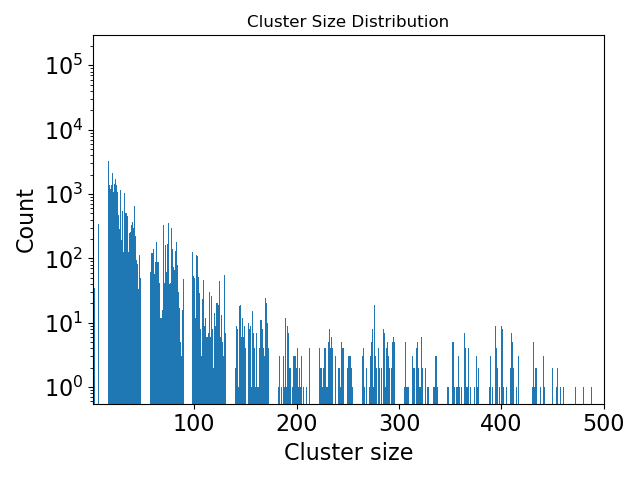

Supplement: Supplementary file 13 — Source data Fig. 8 [file 44320_2025_139_MOESM13_ESM.zip › Figure8/Simulation_software_config_to_run_sim/Metabolon/avg_cluster_count_distribution_second_half.png]
