# Supplementary material for: Conserved interfaces mediate multiple protein–protein interactions in a prokaryotic metabolon
Source: Mol Syst Biol. 2025 Sep 3;21(11):1490–521. doi: 10.1038/s44320-025-00139-9 (PMC12583656; doi:10.1038/s44320-025-00139-9)
Supplement: Supplementary file 14 — Expanded View Figures [file 44320_2025_139_MOESM14_ESM.pdf]

Expanded View Figures

Figure EV1. The original asymmetric matrix shows similar cluster formation as the symmetric matrix in Fig 2.

(**A**) The complete asymmetric matrix of all interactions measured in both configurations (NYFP-A/CYFP-B) and (NYFP-B/CYFP-A). Figure 2A is derived from this matrix by taking the maximum value for each PPI pair. As in Fig. 2A, red boxes indicate MFI > 1100, yellow for 900 < MFI < 1100, blue for 750 < MFI < 900 while white boxes indicate MFI < 750. Gray boxes indicate no measurement. (**B**) Based on the original matrix in (**A**) and Dataset EV2, we calculate the statistical significance of the clusters identified in Fig. 2E. The results are largely similar to Fig. 2E. For each cluster, the red data point represents the median MFI value of the cluster, while the box represents a null distribution generated from the median values of an equal number of interaction pairs (as the cluster size) randomly picked 10,000 times from the matrix. The box represents 25–75 percentile of the data and line in between represents the median of the distribution. The whiskers represent the 5–95 percentile interval. Statistical significance and p value are calculated using a one-sided non-parametric permutation test as described in “Methods.” *** indicates p value < 0.001, ** indicates p value < 0.01. No data normality is assumed. p value = 0.0002 for folate cluster, p value = 0.0001 for purine pathway, p value = 0.0001 for purine cluster, p value = 0.0028 for folate–purine intercluster.

© The Author(s)

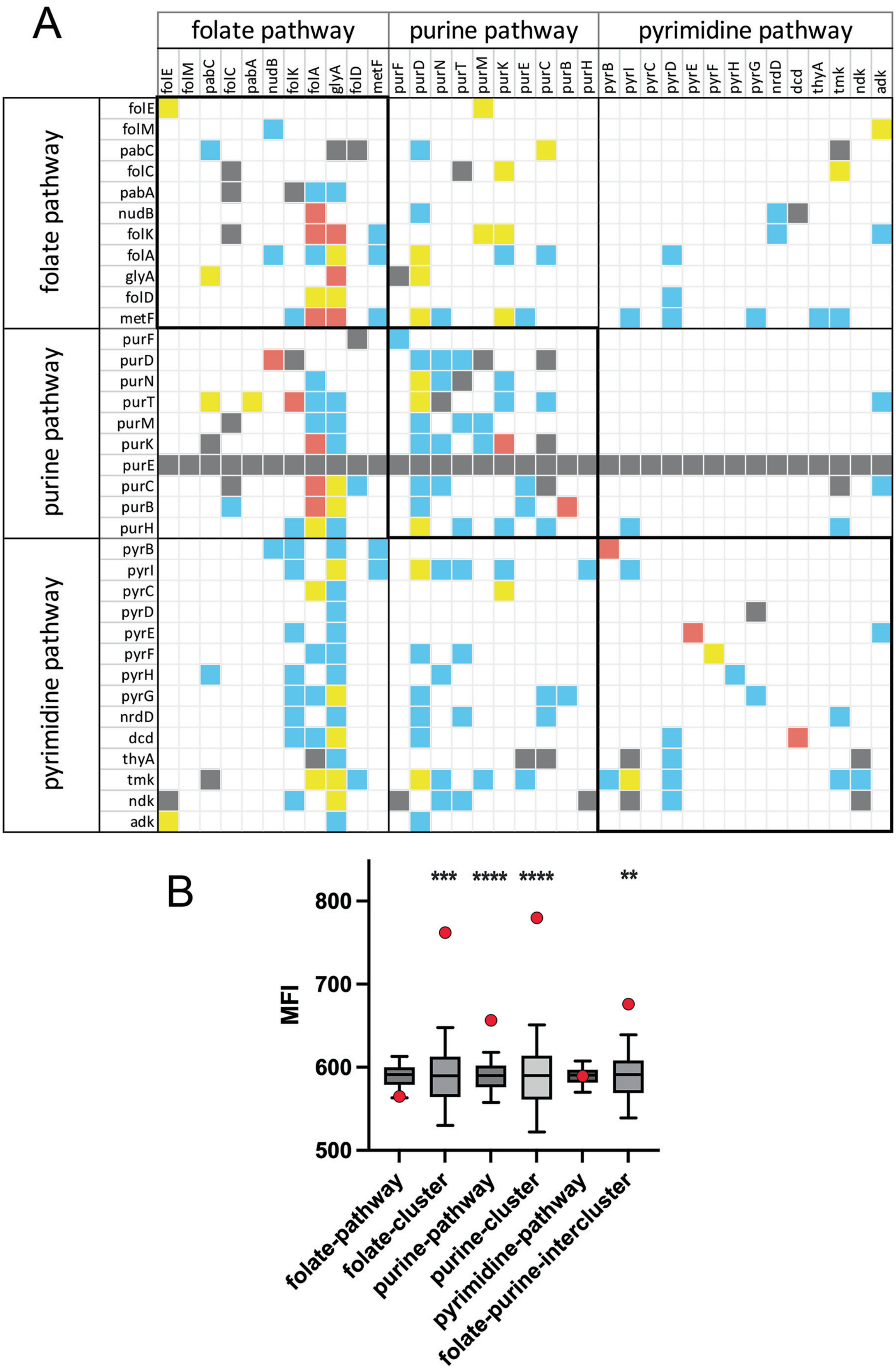
A
folate pathway
purine pathway
pyrimidine pathway
folE
folM
pabC
folC
pabA
nudB
folK
folA
glyA
folD
metF
purF
purD
purN
purT
purM
purK
purE
purC
purB
purH
pyrB
pyrI
pyrC
pyrD
pyrE
pyrF
pyrH
pyrG
nrdD
dcd
thyA
tmk
ndk
adk
B
MFI
800
700
600
500
***
****
****
**
folate-pathway
folate-cluster
purine-pathway
purine-cluster
pyrimidine-pathway
folate-purine-intercluster

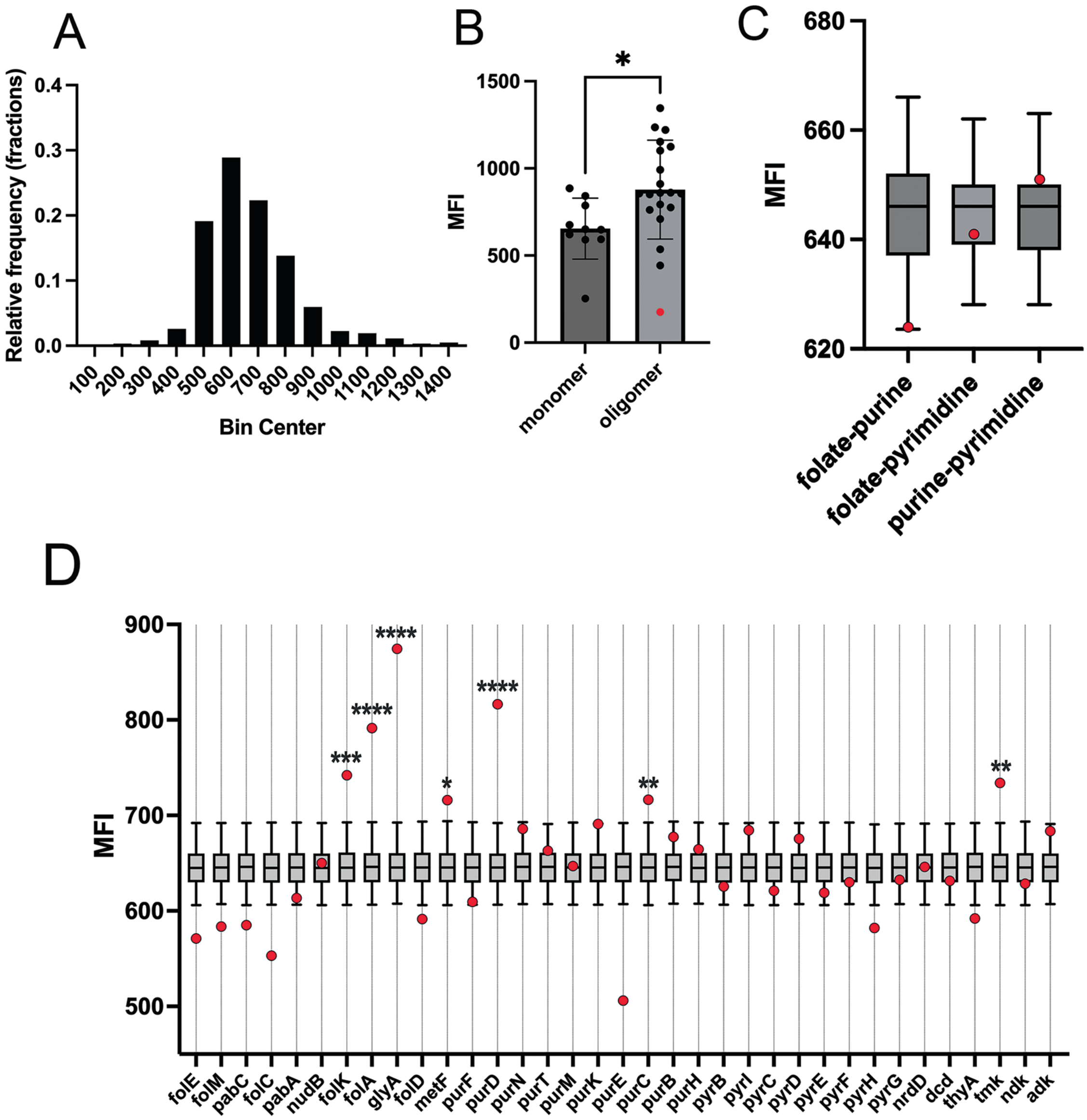

Figure EV2. Cluster formation and interaction propensity of different proteins in the dataset.

(**A**) Frequency distribution of mean YFP fluorescence intensities of 1225 interactions from the dataset. The intensities show a wide range of variation. (**B**) All proteins in our dataset for which oligomeric status is known from crystal structure data (total 30 proteins) were grouped into two classes—monomer and oligomer (dimer and above). The plot shows the distribution of MFI values (homo-oligomer interactions along the diagonal in Fig. 2E) for all proteins belonging to that group. Error bars represent standard deviation. * indicates p value < 0.05, ** indicates p value < 0.01. A two-tailed unpaired t test shows that oligomers have significantly higher fluorescence intensity compared to monomers (p value = 0.03, $n = 10$ for monomer set, and $n = 20$ for oligomer set). The red data point corresponds to PurT, for which the MFI of 175 was much lower than the cellular background fluorescence for non-interacting protein pairs. If this data point is excluded, then the p value becomes strongly significant with p value of 0.005. (**C**) There is no significant inter-pathway interaction among folate, purine, and pyrimidine biosynthesis pathways when entire pathways are taken into consideration. For each cluster, the median MFI value of the cluster (red data point) is compared against a null distribution that is generated from the median values of an equal number of interaction pairs (as the cluster size) randomly picked 10,000 times from the matrix (represented by the box plot, where the box represents 25–75 percentile of the data and line in between represents the median of the distribution. The whiskers represent the 5–95 percentile interval). Statistical significance and p value are calculated using a one-sided non-parametric permutation test as described in "Methods." No data normality is assumed. (**D**) Interaction propensity of each protein with all other proteins in the dataset, represented as the average of MFI across all 35 proteins in the dataset. The median (red data point) of all 34 interactions of a protein (except with itself) is compared against a null distribution, as explained in (**C**). Cases for which the p values were significant are shown with *. Overall, **** indicates p value < 0.0001, *** indicates p value < 0.001, ** indicates p value < 0.01, while * indicates p value < 0.05. The actual p values were the following: FolK—0.0008, FolA—0.0001, GlyA— 0.0001, MetF—0.01, PurD—0.0001, PurC—0.0076, Tmk—0.0022. Statistical significance and p value are calculated using a one-sided non-parametric permutation test as described in "Methods." No data normality is assumed. For box-plots, the box represents 25–75 percentile of the data and line in between represents the median of the distribution. The whiskers represent the 5–95 percentile interval.

 © The Author(s)

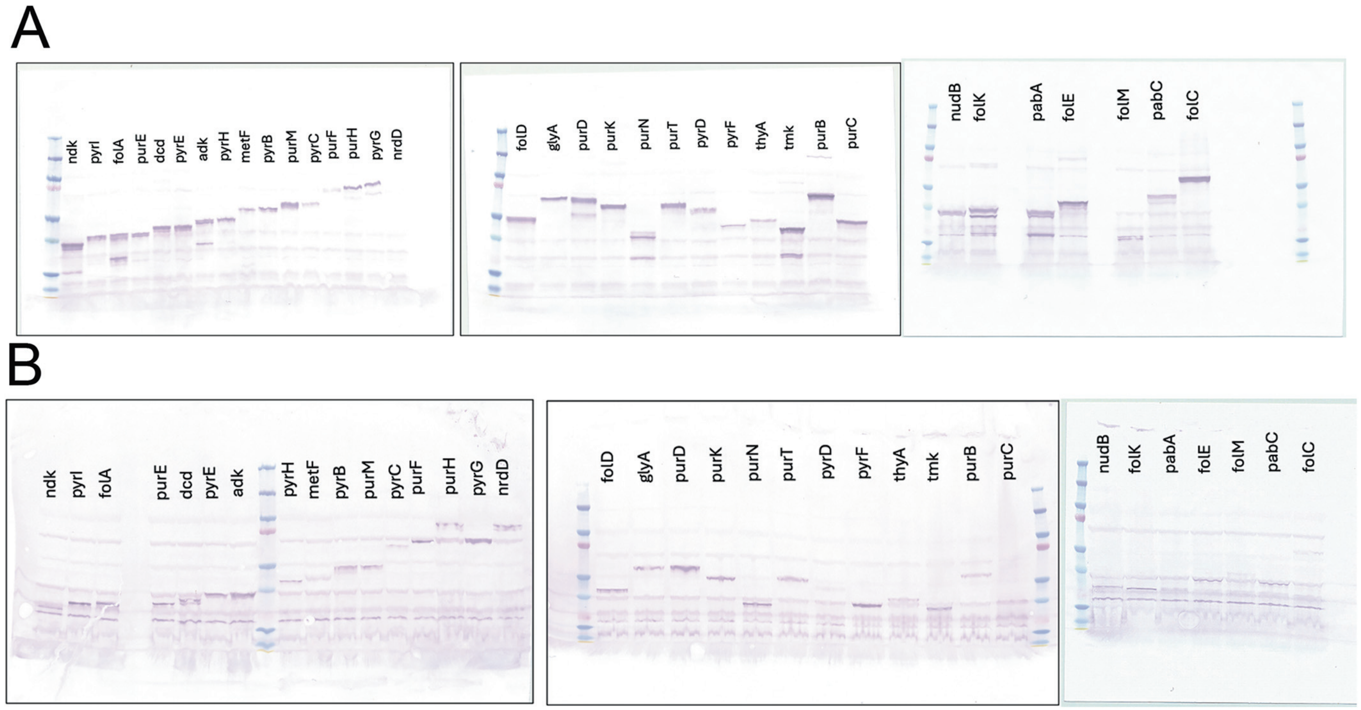

Figure EV3. Expression check for different NYFP and CYFP fusion constructs.

Western blot images to check the expression of (**A**) NYFP- and (**B**) CYFP- fusion constructs using polyclonal anti-YFP antibody (see "Methods" for details). Since the NYFP fragment is larger, NYFP-fusion proteins have higher intensities on the blot than their CYFP counterparts, and therefore, blot (**B**) shows significantly more background. In all cases, the most prominent band that matches the expected molecular weight of the fusion proteins was used for quantification. Overall, though expression levels of the fusion proteins vary, they are not sufficient to explain differences in observed PPI strengths.

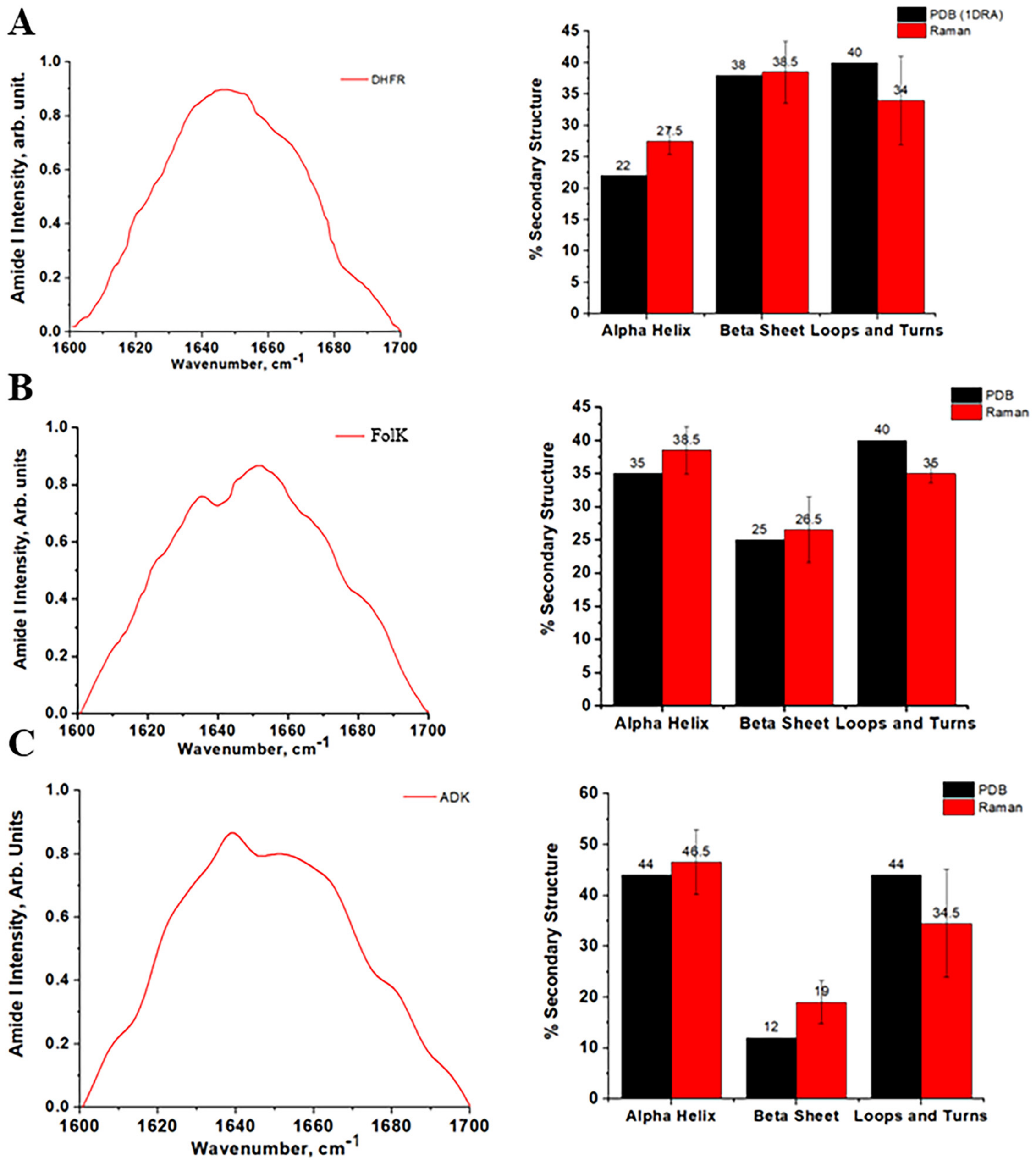

Figure EV4. Comparison of secondary structure content from Raman spectra and the PDB structure.

(**A**) Amide I (1600–1700 cm^{-1}) Raman spectral profile of WT DHFR. The plot shown is the mean of the acquisition from three independent technical repeats. The bar plot shows the secondary structure contents of DHFR (black bars) as obtained upon deconvolution of the Amide I spectrum. Amide I spectra for DHFR were deconvoluted using Lorentzian peak fitting and the secondary structural contents for helix, beta sheet, turns, and loops were compared with the solved structure (black bars, PDB 1DRA). Peaks were selected based on the standard Amide peaks used for secondary structure analysis, and the quality of the fit was checked using the reduced chi-squared value, which was typically between 1.2 and 1.5. The secondary structural content was found to be comparable to the PDB structure, further reflecting the quality of the spectra and the peak fitting carried out. (**B**) A similar analysis was done for FolK. The secondary structural content was found to be comparable to the PDB structure. (**C**) Amide I spectral profile of the control protein ADK was acquired. A similar analysis was done for ADK, and the secondary structural content was found to be comparable to the PDB structure. $n = 3$ biologically independent samples were used for the experiments. All the data are presented as mean values +/− SEM.

 © The Author(s)

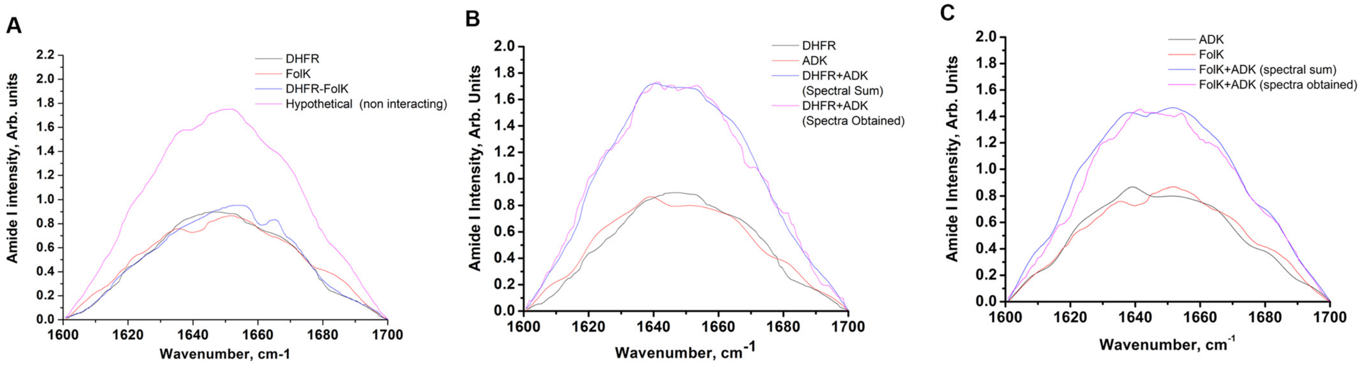

Figure EV5. Raman spectra of DHFR, ADK and FolK proteins alone and in complex.

(**A**) Raman spectra obtained from DHFR–FolK interaction set do not overlap with the hypothetical spectrum, which is the mathematical sum of DHFR and FolK spectra. Raman spectra for control sets using ADK and each of DHFR (**B**) and FolK (**C**) were acquired using laser excitation of 785 nm. Intensities (arbitrary units, Arb. Units.) for native DHFR and ADK in the amide I range (1600–1700 cm^{-1}) were recorded individually and then upon mixing. The resultant spectra obtained were found to be comparable to the mathematical sum of the individual spectrum of DHFR and ADK, potentially suggesting the absence of any interaction. (**C**) The resultant spectrum was found to be comparable to the mathematical sum of the individual spectrum for ADK and FolK, which suggests the absence of any interaction. $n = 3$ biologically independent samples were used for the experiments. All the data are presented as mean values +/− SEM.

© The Author(s)

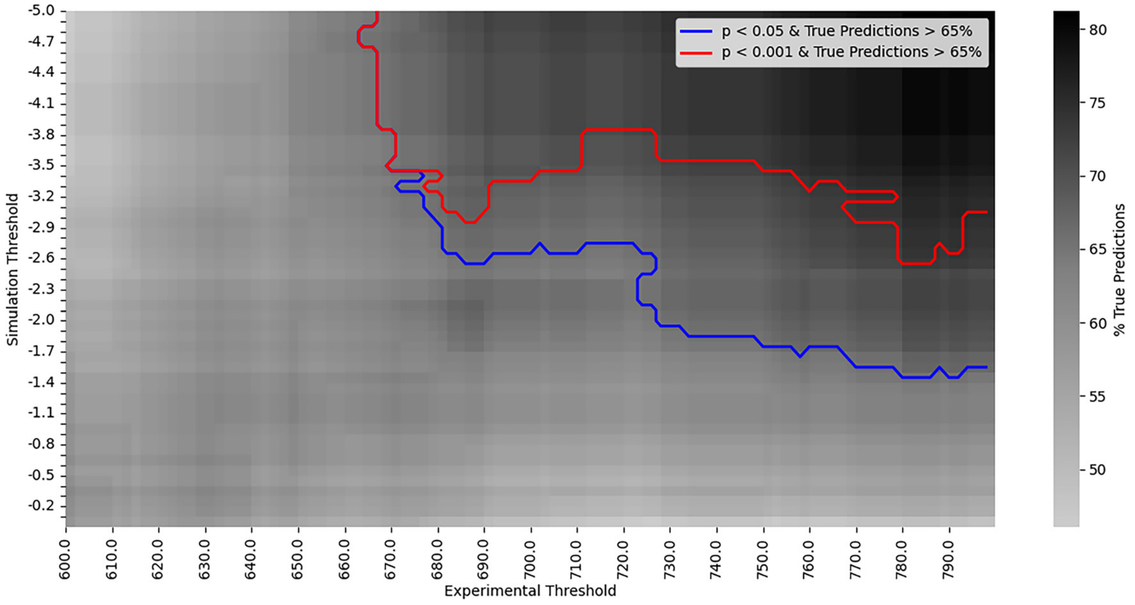

Figure EV6. Robustness of simulation predictions.

The sensitivity of true predictions % according to simulation to varying thresholds of $\Delta G_{binding}$) and the experimental threshold value from fluorescence experiments (*x*-axis). The regions within the blue and red contours are statistically significant at *p* values of <0.05 and <0.001, respectively. The combination of thresholds within the shaded regions results in statistically significant and strong agreement, wherein >65% of stable binding predictions from simulations match that of experiments.

 © The Author(s)

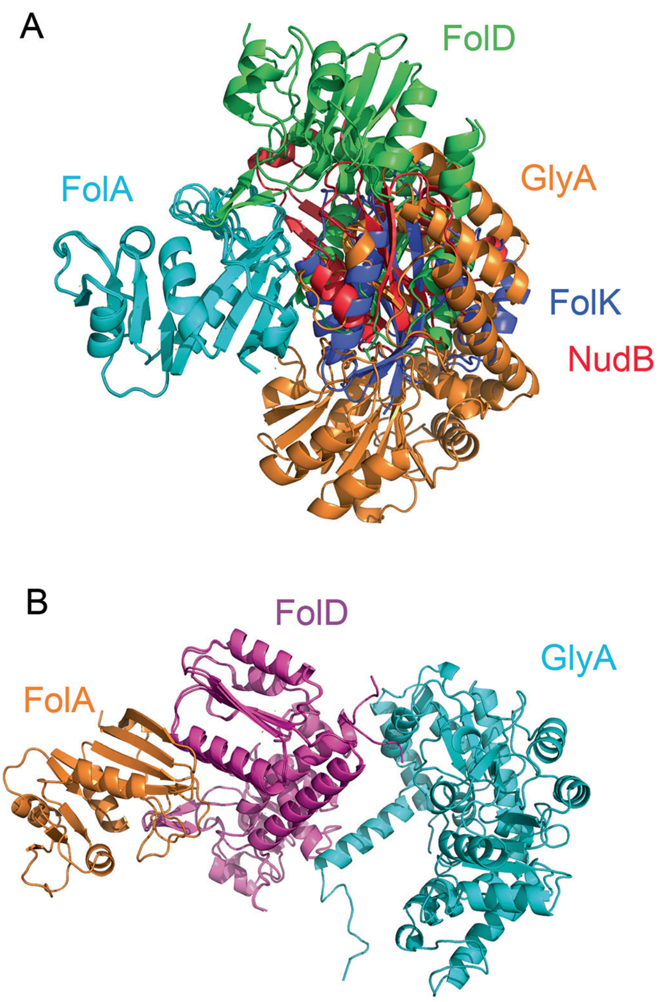

Figure EV7. Analysis of complex structures predicted by Alphafold.

(**A**) Structural alignment of PPI complexes of FolA with different binding partners (FolD, GlyA, FolK, and NudB) shows that FolA uses a similar interface to interact with multiple proteins, hence the highly significant *p* value of FolA (p value = 1.2e−9 in Fig. 7B). (**B**) Structural alignment of PPI complexes of FolD with two different binding partners (FolA and GlyA) shows that the two interfaces are completely different, hence the non-significant *p* value (p value = 0.279 in Fig. 7B).

© The Author(s)

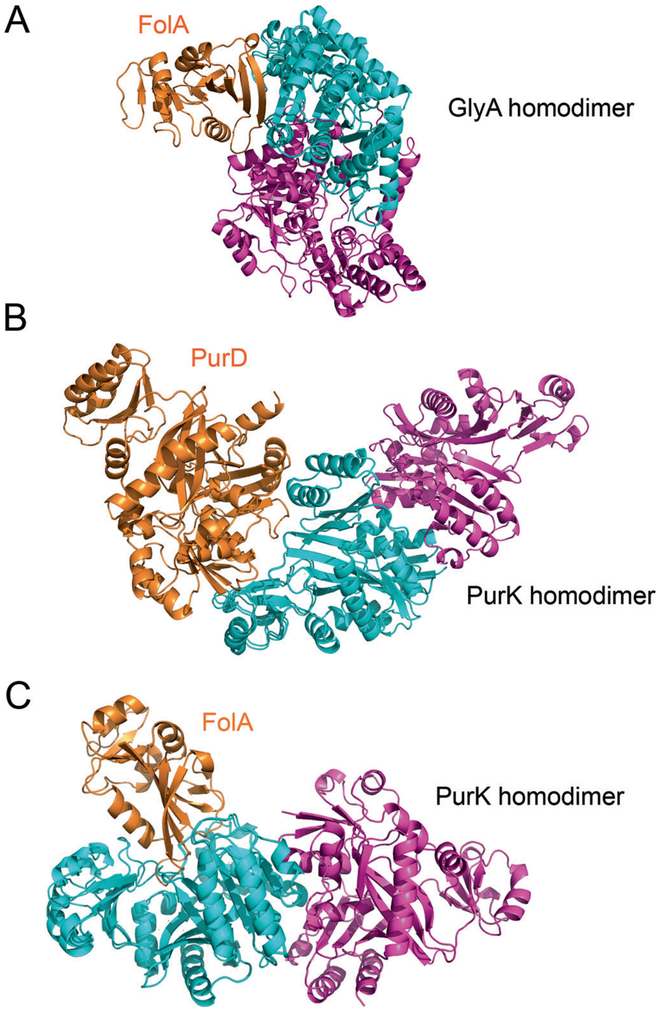

Figure EV8. Overlay of representative heterodimeric structures of PPI complexes with their corresponding homodimeric structures.

(**A**) FolA-GlyA complex superimposed with GlyA homodimer, (**B**) PurD-PurK complex superimposed with FolK homodimer, and (**C**) FolA-PurK complex superimposed with FolK homodimer. In all these structures, the homodimeric interface is different than the PPI interface.

 © The Author(s)

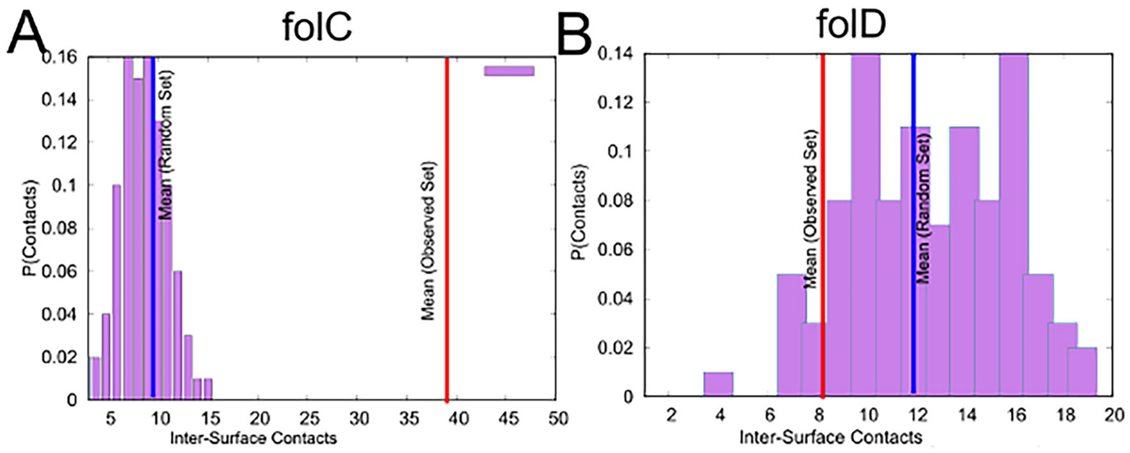

Figure EV9. Proteins use similar interface to interact with multiple other proteins in the pathway.

Distribution of mean pairwise inter-surface contacts for a set of 50 randomly drawn surfaces on (**A**) FolC and (**B**) FolD proteins. The distribution corresponds to the mean inter-surface contacts for 10,000 such realizations, each with 50 randomly drawn surfaces. The blue horizontal line shows the mean value for inter-surface contacts for the random set, while the red line shows the corresponding value for the observed surfaces. (**A**) For the FolC protein, the mean inter-surface contacts (observed) is significantly higher than the corresponding value for the random set, suggesting that the surfaces used for interaction across different proteins share a high degree of overlap. (**B**) On the other hand, the interaction surfaces on FolD corresponding to different interaction partners show no such overlap.

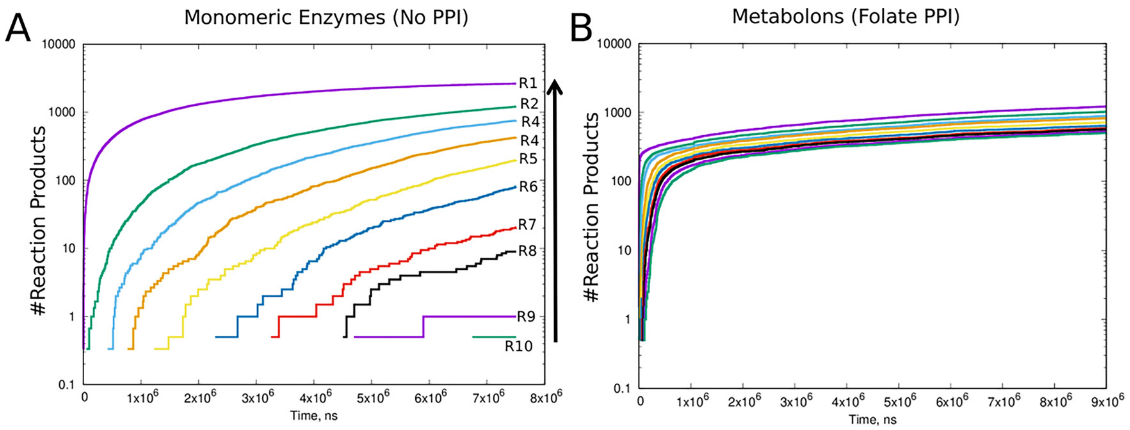

Figure EV10. Protein-protein interactions significantly accelerate reactions compared to a diffusion controlled model.

Progression of the ten reactions in the diffusion-reaction model during simulation for (**A**) monomeric enzymes where all reactions occur in the bulk and (**B**) when enzymes interact via a PPI map based on experiments.

 © The Author(s)
